# Genome-Wide Identification and Expression Analysis of AP2/ERF Transcription Factor Related to Drought Stress in Cultivated Peanut (*Arachis hypogaea* L.)

**DOI:** 10.3389/fgene.2021.750761

**Published:** 2021-10-13

**Authors:** Mengjie Cui, Muhammad Salman Haider, Pengpei Chai, Junjia Guo, Pei Du, Hongyan Li, Wenzhao Dong, Bingyan Huang, Zheng Zheng, Lei Shi, Xinyou Zhang, Suoyi Han

**Affiliations:** ^1^ College of Agriculture, Nanjing Agricultural University, Nanjing, China; ^2^ Henan Institute of Crop Molecular Breeding, Henan Academy of Agricultural Science/Key Laboratory of Oil Crops in Huang-Huai-Hai Plains, Ministry of Agriculture/Henan Provincial Key Laboratory for Oil Crops Improvement, Zhengzhou, China; ^3^ Department of Horticulture, Ghazi University, Dera Ghazi Khan, Pakistan

**Keywords:** AP2/ERF1, peanut, phylogenetic analysis, evolution analysis, drought stress

## Abstract

APETALA2/ethylene response element-binding factor (AP2/ERF) transcription factors (TFs) have been found to regulate plant growth and development and response to various abiotic stresses. However, detailed information of *AP2/ERF* genes in peanut against drought has not yet been performed. Herein, 185 AP2/ERF TF members were identified from the cultivated peanut (*A. hypogaea cv.* Tifrunner) genome, clustered into five subfamilies: AP2 (APETALA2), ERF (ethylene-responsive-element-binding), DREB (dehydration-responsive-element-binding), RAV (related to ABI3/VP), and Soloist (few unclassified factors)). Subsequently, the phylogenetic relationship, intron–exon structure, and chromosomal location of *AhAP2/ERF* were further characterized. All of these *AhAP2/ERF* genes were distributed unevenly across the 20 chromosomes, and 14 tandem and 85 segmental duplicated gene pairs were identified which originated from ancient duplication events. Gene evolution analysis showed that *A. hypogaea cv.* Tifrunner were separated 64.07 and 66.44 Mya from *Medicago truncatula* L. and *Glycine max* L., respectively. Promoter analysis discovered many *cis*-acting elements related to light, hormones, tissues, and stress responsiveness process. The protein interaction network predicted the exitance of functional interaction among families or subgroups. Expression profiles showed that genes from *AP2*, *ERF*, and *dehydration-responsive-element-binding* subfamilies were significantly upregulated under drought stress conditions. Our study laid a foundation and provided a panel of candidate AP2/ERF TFs for further functional validation to uplift breeding programs of drought-resistant peanut cultivars.

## Introduction

Transcription factors (TFs) (or *trans*-acting factors) are the main class of regulatory proteins that can specifically combine with DNA-binding domains and perform a key role by regulating the expression of downstream genes (Singh et al., 2002; Licausi et al., 2013). Nearly 60 different TF families have been found in higher plants, such as AP2/ERF (Gutterson and Reuber, 2004; Xu et al., 2011; Li M.-Y. et al., 2015), ARF (Finet et al., 2010; Rademacher et al., 2011), bHLH (Li et al., 2006), bZIP (Ulm et al., 2004; Liu et al., 2012), C2H2 (Tsutsui et al., 2011), MADS (Trevaskis et al., 2003; Terol et al., 2019), MYB (Dubos et al., 2010; Feller et al., 2011), NAC (Mao et al., 2012; Nakashima et al., 2012), SBP (Kandori et al., 2006; Ferreira et al., 2014), and WRKY (Rushton et al., 2010). Among these TFs, the APETALA2/ethylene-responsive element-binding factor (AP2/ERF) superfamily contains the largest group of TFs in plant, which are reportedly involved in plant growth progress and abiotic stress responsiveness according to relevant reports (Licausi et al., 2013; Feng et al., 2020). The first AP2/ERF TF was found to regulate flower development in *Arabidopsis* (Jofuku et al., 1994). Subsequently, AP2/ERF genes were widely found in leaf, root, seed, fruit, and other tissues (Chuck et al., 2002; Hirota et al., 2007; El-Sharkawy et al., 2009; Pietsch et al., 2009; Kitomi et al., 2011; Soares et al., 2011). Not only in plants, related AP2/ERF superfamily proteins are also found in ciliates and protists that may be associated with the His- and Asn-rich HNH class of homing endonucleases (Magnani et al., 2004; Wuitschick et al., 2004).

AP2/ERF TFs usually contain one or two AP2-conserved domains (60–70 amino acid residues) which combine with the *cis*-acting elements in the promoter regions of targeted genes (Okamuro et al., 1997; Allen et al., 1998; Riechmann and Meyerowitz, 1998). The *AP2/ERF* superfamily genes are mainly divided into AP2 (APETALA2), DREB (dehydration-responsive-element-binding), ERF (ethylene-responsive-element-binding), RAV (related to ABI3/VP), and Soloist (few unclassified factors) subfamilies based on the sequence characteristics and the number of AP2-conserved domains (Nakano et al., 2006; Tamura et al., 2011). In most cases, the AP2 subfamily contains proteins with two AP2 domains involved in regulating plant developmental processes (El et al., 2010). ERF, DREB, and RAV subfamily members contain only one single AP2 domain, while RAV members are often associated with an additional B3 DNA-binding domain (Licausi et al., 2010). Discrepancy of 14th and 19th amino acid sequences is the main differences between ERF and DREB subfamilies; the ERF subfamily consists of alanine (Ala) and aspartate (Asp) whereas the DREB subfamily consists of valine (Val) and glutamic acid (Elu) of 14th and 19th amino acid sequences, respectively (Sakuma et al., 2002). Additionally, other members with special gene structure and AP2-like domain are known as Soloist ones (Li H. et al., 2017).

With more draft genomic information of plants released, AP2/ERF superfamily members have been identified and characterized in eudicots, i.e., *Arabidopsis* (Sakuma et al., 2002; Nakano et al., 2006), grapevine (Licausi et al., 2010), cucumber (Hu and Liu, 2011), Chinese plum (Du et al., 2013), apple (Girardi et al., 2013), sweet orange (Ito et al., 2014), pineapple (Huang et al., 2020), canola (Ghorbani et al., 2020), Chinese cherry (Zhu et al., 2021), and dark jute (Kabir et al., 2021), and in monocots, i.e., rice (Sharoni et al., 2011), common wheat (Zhuang et al., 2011), sugarcane (Li et al., 2020), maize (Liu et al., 2013), barley (Guo et al., 2016), and foxtail millet (Lata et al., 2014). In general, AP2 TFs have been found to regulate various developmental processes, such as the development of floral organs (Irish and Sussex, 1990; Jofuku et al., 1994; Chuck et al., 1998 and, 2008; Maes et al., 2001; Aukerman and Sakai, 2003) and embryo and seed growth (Boutilier et al., 2002; Jofuku et al., 2005; Krizek and Beth, 2009). ERF and DREB subfamily proteins mainly function in the resistance to diverse biological and environmental stresses, such as biotic stresses (microbial pathogens and herbivorous insects) and abiotic stresses (drought, heat, cold, and salinity) (Feng et al., 2020). Additionally, RAV subfamily proteins play a crucial role against biotic and abiotic stress responses (Sohn et al., 2006; Li et al., 2011; Fu et al., 2014) by responding to the signal of plant hormones (ethylene and brassinosteroid) (Alonso et al., 2003; Hu et al., 2004).

Peanut, an important oil and economic crop worldwide, is used to provide oil and proteins for humans (Zhuang et al., 2019). In particular, with the characteristics of underground fruit, its yield is being devastatingly affected by drought stress (Vahdati and Lotfi, 2013). Additionally, drought will also result in the increase of aflatoxin contamination and the frequency of diseases and pests (Boyer, 1982; Nimitr et al., 2003). To date, drought has been the most serious abiotic stress which negatively affects the quality and distribution of peanut (Reddy et al., 2003; Farombi, 2006; Cardwell and Henry, 2008; Sun et al., 2013; Jayaprakash et al., 2019; Soni et al., 2020). Previous studies have demonstrated the critical role of *AP2/ERF* genes in mediating drought stress resistance. For example, AhERF3 and AhERF5 in root were upregulated by PEG treatment (Chen et al., 2012), indicating its association with drought stress. Notably, overexpression of *AhERF019* could enhance tolerance to drought in transgenic *Arabidopsis* (Wan et al., 2014). Thus, there may be more important *AP2/ERF* members that act in enhancing the resistance to drought stress of peanut. In recent years, with the release of genome information with cultivated peanut (*A. hypogaea cv.* Tifrunner), an allotetraploid (AABB 2n = 4x = 40), and related wild-type ones (diploids: *A. duranensis* and *A. ipaensis*) (Bertioli et al., 2016; Zhuang et al., 2019). Several gene families, including monosaccharide transporter MST genes (Wan et al., 2020), GRF (Zhao et al., 2019), bHLH (Chao et al., 2017), and WRKY (Song et al., 2016), have been characterized at a genome scale. However, very limited information of *AP2/ERF* genes is available in cultivated peanut.

In the present study, 185 AP2/ERF superfamily members of *A. hypogaea cv.* Tifrunner were investigated by phylogenetic relationship, sequence structures, chromosomal distributions, duplication events, and promoter region analysis. The expression patterns of *AhAP2/ERF* under drought stress were quantified by using quantitative real-time polymerase chain reaction (qRT-PCR). Our investigation will be beneficial to identify the drought-responsive candidate genes for further functional characterization to breed drought-resistant peanut cultivars.

## Materials and Methods

### Screening and Identification of the *AP2/ERF* Superfamily Genes

To accurately collect all members of *AhAP2/ERF* genes and avoid nonspecific amplification, multiple-database searches were performed. The *A. hypogaea cv.* Tifrunner genome sequences were downloaded from peanutbase (https://www.peanutbase.org/). The AP2 domain (PF00847) profile was obtained from the pfam database (http://pfam.janelia.org), which was used to match each member of AP2/ERF protein in genomes using HMMER 3.1 software (E-value<1e^−5^) (Finn et al., 2011). To avoid the omission of AP2/ERF members, we also performed searches in the Transcription Factor database (http://planttfdb.cbi.pku.edu.cn/) (Jin et al., 2014). All protein sequences acquired were then verified for the AP2 domain by using the SMART (Simple Modular Architecture Research Tool: http://smart.embl-heidelberg.de/) (Letunic et al., 2012) and Pfam (http://pfam.xfam.org) databases (Finn et al., 2008) and NCBI (https://www.ncbi.nlm.nih.gov/cdd). Proteins lacking compete AP2 domains were identified by manual examination. Physichochemical profiling of *AP2/ERF* genes was performed by using online ExPASy (Gasteiger, 2005; Panu et al., 2012). The subcellular localization analysis of curated *AP2/ERF* superfamily genes was conducted on the Plant-Ploc server (http://www.csbio.sjtu.edu.cn/bioinf/plant/) (Chou and Shen, 2008).

### Phylogenetic Analysis of AP2/ERF Proteins

The AP2 domains were extracted based on results of SMART (Simple Modular Architecture Research Tool: http://smart.embl-heidelberg.de/) (Letunic et al., 2012). Multiple-sequence alignment was executed by DNAMAN and CLUSTAL program (Thompson et al., 1994; Burner and Legendre, 2000). To construct phylogenetic trees, MEGA 7.0 software was used with the neighbor-joining model (1,000 replicates) (Tamura et al., 2013). AP2/ERF family gene names in *A. hypogaea cv.* Tifrunner were given according to the ascending order of location on chromosomes.

### Gene Structure and Conserved Motif Analysis

Information of the intron–exon structure was obtained from the reference peanut genome (*A. hypogaea cv.* Tifrunner, https://www.peanutbase.org/). The Multiple Expectation Maximization for Motif Elicitation program (MEME, http://meme-suite.org/tools/meme) was used to identify potential conserved motifs shared by 185 *AhAP2/ERF* genes (Bailey et al., 2009). Basic information extraction and preliminary drawing of sequence structure were conducted using TBtools (South China Agricultural University, Guangdong, China) (Chen et al., 2020).

### Chromosome Localization, Duplications, and Evolutionary Analysis of *AhAP2/ERF*

MapChart 2.3 software developed by Wageningen University and Research in Wageningen, Netherlands, is used to locate genes on chromosomes (Voorrips, 2002). Tandem and segmental genes, Ka/Ks values, and circos figures for chromosome locations with AP2/ERF duplication links were completed by TBtools software (South China Agricultural University, Guangdong, China) (Chen et al., 2020). Duplication and divergence time were calculated by the following formula as described by Bertioli et al. (2016):

$$\text{T}=\text{Ks}/2\text{λ}\left(\text{λ}=8\text{.}12\times 10^{-9}\right)$$

### Promoter Analysis of *AhAP2/ERF* Genes

Approximately 1,500-bp upstream sequences of the *AhAP2/ERF* genes were used to get a better knowledge of the potential function of promoter. PlantCARE (http://bioinformatics.psb.ugent.be/webtools/plantcare/html/) (Lescot et al., 2002) was used to identify the *cis*-regulatory elements exited in the gene promoters related to stress responses and hormone effects, and these results were visualized by TBtools.

### Prediction of the Protein Interaction Network

Prediction of the protein interaction network was conducted on the basis of the STRING database (https://string-db.org/. accessed on January 28, 2021) (Szklarczyk et al., 2019). *Arabidopsis thaliana* L., the well-characterized model plant, was the subject organism (combined score≥ 0.4). PPI networks were constructed by Cytoscape software v 3.8.0 (Shannon et al., 2003).

### Plant Materials and Drought Stress Treatment

Seeds of *Arachis hypogaea* L., ‘YUANZA 9102’, laboratory homozygous material, were sown in 392 cm^3^ (7 cm long, 7 cm wide, and 8 cm high) pots which were filled with a mixture of vermiculite and perlite (3:1 v/v). Plants were put in a fully controlled growth room (relative humidity: 70%, 16 h/8 h light/dark; 30°C/28°C day/night; light intensity 17,000 lx). Watering was stopped in one part of pots (drought treatment) when seedlings were 5 weeks old, whereas the watering regime remained unchanged in the control plants (every 4 days). Roots were collected from the control and treatment groups every 4 days from seedlings aged 5–9 weeks. All samples were frozen in liquid nitrogen immediately and then stored at -80°C for RNA isolation.

### RNA Isolation and Quantitative RT-PCR Assays

RNAprep Pure Plant Plus Kit (Tiangen Biotech, Co., Beijing, China) was utilized to extract RNA from control and treated peanut samples. The cDNA was prepared by following the user manual of the PrimeScrit™ RT Kit with gDNA eraser (perfect real-time, Takara Biomedical Technology, Ltd., Beijing, China). Thirty-five genes from each family were designed by Primer Premier 5.0 and are shown in Supplementary Table S1. The alcohol dehydrogenase class III (*AhADH3*, *Arahy. VYWU26.2*) (forward primer: 5′-GAC​GCT​TGG​CGA​GAT​CAA​CA-3′, reverse primer: 5′-AAC​CGG​ACA​ACC​ACC​ACA​TG-3′) was selected as the internal reference control (Brand and Hovav, 2010). Subsequently, quantitative RT-PCR was performed using the ABI QS5 qRT-PCR detection system (ABI, United States) and SYBR Green Kit (Tiangen, Beijing, China). An ABI QS5 real-time PCR system was used under the following procedure: 95°C for 15 min, followed by 40 cycles of 95°C for 10 s, and 60°C for 32 s in a 20 µl volume. Each PCR assay was carried out in three biological replicates, of which each replicate corresponded to three technical repeats. Relative expression levels of the genes were calculated using the 2^-△△Ct^ method (Livak and Schmittgen, 2001).

## Results

### Characterization and Phylogenetic Analysis of AhAP2/ERF Family Proteins in Cultivated Peanut

**FIGURE 1:**
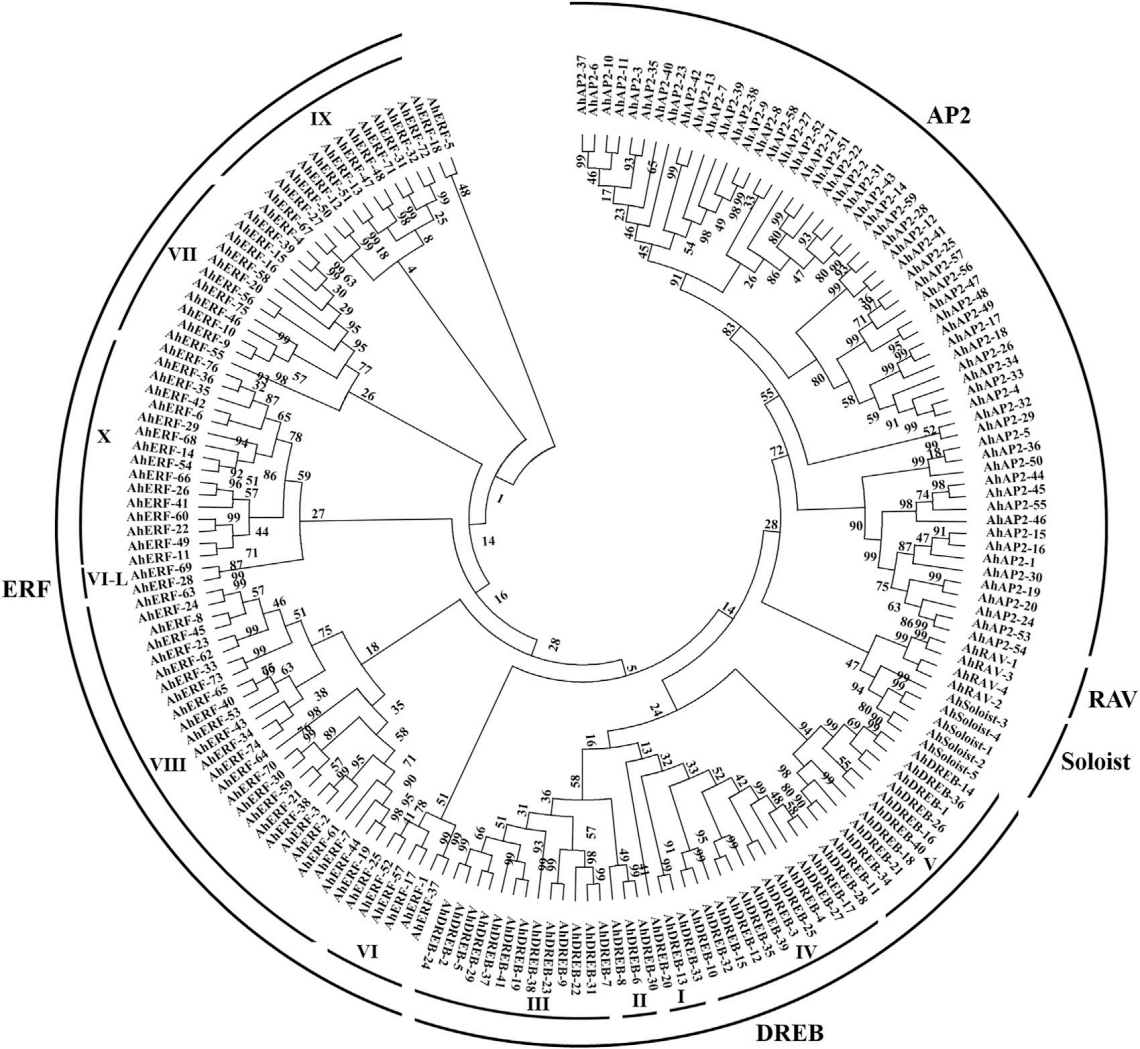
Phylogenetic tree of AP2/ERF TFs in *A. hypogaea* cv. Tifrunner.

A total of 185 unigenes with the AP2 domain were characterized as AP2/ERF TFs in *A. hypogaea cv.* Tifrunner (Supplementary Table S2). Depending on the sequence characteristics, the AP2 domains, phylogenetic tree analysis, and the classification system established by the group of Yamaguchi-Shinozaki (Tamura et al., 2011) and Shinshi (Nakano et al., 2006), the *AP2/ERF* superfamily genes are mainly classified into AP2 (APETALA2), ERF (VI-X, ethylene-responsive-element-binding), DREB (I-V, dehydration-responsive-element-binding), RAV (related to ABI3/VP), and Soloist (few unclassified factors) subfamilies (Figure 1 and Supplementary Figure S1). Among these, the 27 to encode two AP2 domains and the 4 to one AP2 domain together with one B3 domain were thus assigned to the AP2 and RAV families, respectively. Based on the similarity of amino acid sequences with the AP2 domain, the 117 genes were further assigned to the ERF 76) and DREB 41) subfamilies, respectively. Thirty-two members with a single AP2 domain but were distinct from the ERF or DREB subfamily were classified into the AP2 subfamily (Supplementary Table S2). The remaining five genes with independent clades from others were identified as *Soloist* genes. Subsequently, all superfamily members were named according to the order on the chromosomes of each family member to distinguish from each other for the study (Supplementary Table S3).

To evaluate the phylogenic relationship and classification of the ERF and DREB subfamily, multiple-alignment analyses was performed on the protein sequences with the AP2 domain acquired from peanut (117), *Arabidopsis* (139), and rice (139), as suggested by Nakano et al. (2006) (Supplementary Figures S2, S3). The NJ phylogenetic tree divided the ERF and DREB subfamilies of peanut and Arabidopsis into 10 subgroups (DREB-I-V and ERF-VI to X) following the classification as described by Nakano et al. (2006) (Supplementary Figure S4). The phylogenetic tree of ERF and DREB subfamily proteins of peanut and rice also exhibited similar results (Supplementary Figure S5). Overall, current findings of the phylogenetic tree demonstrated that classification of the peanut ERF and DREB subfamily proteins is similar to the *Arabidopsis* and rice ERF family (Supplementary Table S4).

Molecular property analysis showed that MW of *AP2/ERF* superfamily members varied from 14.46 to 82.54 kDa. Most of *AP2/ERF* superfamily genes showed their localization in the nucleus. Moreover, the negative GRAVITY values suggested the globular hydrophilic nature of the AP2/ERF proteins. Interestingly, the members of the same families or clades shared similar physical properties, indicating the functions conservatively in the same clades and differentially among subfamilies.

### Structure Analysis of *AhAP2/ERF*

**FIGURE 2:**
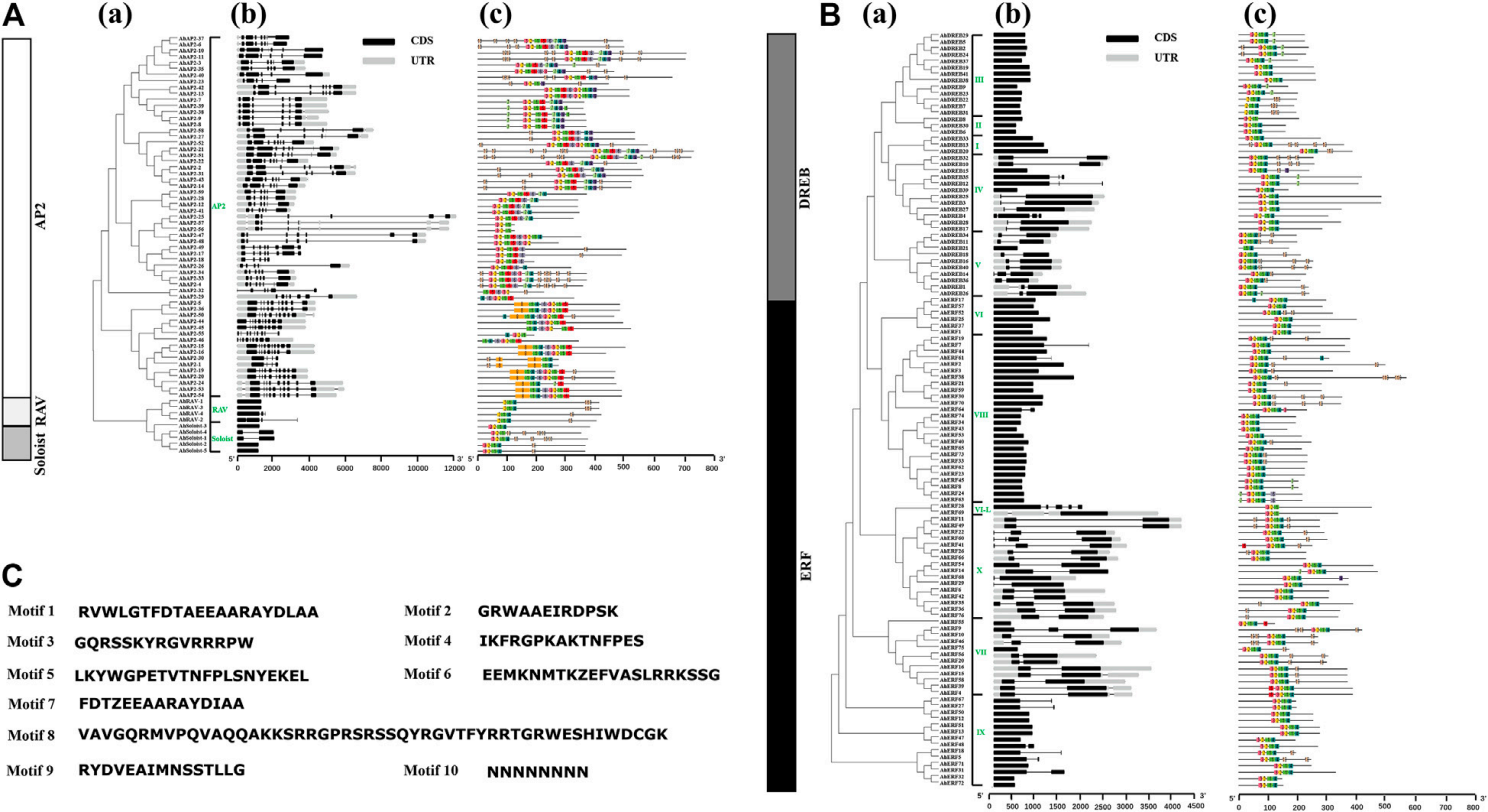
Intron–exon structures and conserved motif analysis of *AhAP2/ERF* genes according to the phylogenetic relationship. **(A)** Related information of AP2, RAV, and Soloist subfamily. **(B)** Related information of ERF and DREB subfamily. **(C)** The amino acid composition of each motif. **(a)** The phylogenetic tree. **(b)** The exon–intron structure of *AP2/ERF* genes. **(c)** The distribution of conserved motifs in AP2/ERF proteins. Each conserved motif is represented by various-colored rectangles. Box length corresponds to motif length. Color blocks of different colors represent different family and group members.

Structural analysis of *AP2/ERF* genes is helpful for us to fully understand the conservative characteristics of peanut AP2/ERF protein and analyze its evolutionary differences. Numbers of introns varied among AP2/ERF subfamilies (Figure 2). All the *AP2* family genes contain 3–10 introns, whereas most members of *ERF*, *RAV*, and *Soloist* subfamilies have only 1–2 introns or do not possess introns. A similar phenomenon was discovered in *Arabidopsis thaliana* L. and *Cucumis sativus* L., where most *Arabidopsis* genes of the ERF family do not possess introns (Sakuma et al., 2002), and 83% of *CsERF* genes do not have intron (Ito et al., 2014). What is more, members of the ERF family clustered into one branch have a similar gene structure (Figure 2). There was some subgroup specificity: members of groups DREB-III, DREB-II, DREB-I, and ERF-VI did not possess introns, and the genes owning two introns were in the groups ERF-VII and ERF-X, possibly attributed to number changes of introns during evolution, whereas the number and position of the introns were relatively conserved in the same group of plant species.

To provide evidence for the classification and the functional conversation of *AP2*/*ERF* superfamily genes among different groups, 10 conserved motifs (motifs 1–10) were analyzed by using MEME software (Figure 2). Among these, motifs 1 and 2 were dominantly present in the AP2 domain regions of all family members. The proteins of the same group showed identical numbers and arrangements of motifs, which are different among the various clades. For example, nine motifs were detected in AP2 and ERF families, and four motifs in RAV and Soloist families. Furthermore, the number and arrangement of motifs in the RAV (motifs 2, 1, 4, 10), ERF, and Soloist (motifs 3, 2, 1, 4) families showed high similarity. Motif 8 was only detected in few AP2 family members, signifying the meticulousness of the motif in the AP2 family. Remarkably, motifs in the same group showed great similarity, indicating the functional conservation in different groups. Comparing the intron–exon structure and conserved motif analysis, it is clear that the members of the same group showed great similarity of characteristics, indicating that most of *AhAP2/ERF* genes were highly conserved among groups.

### Genome Distribution of *AP2/ERF* Genes

**FIGURE 3:**
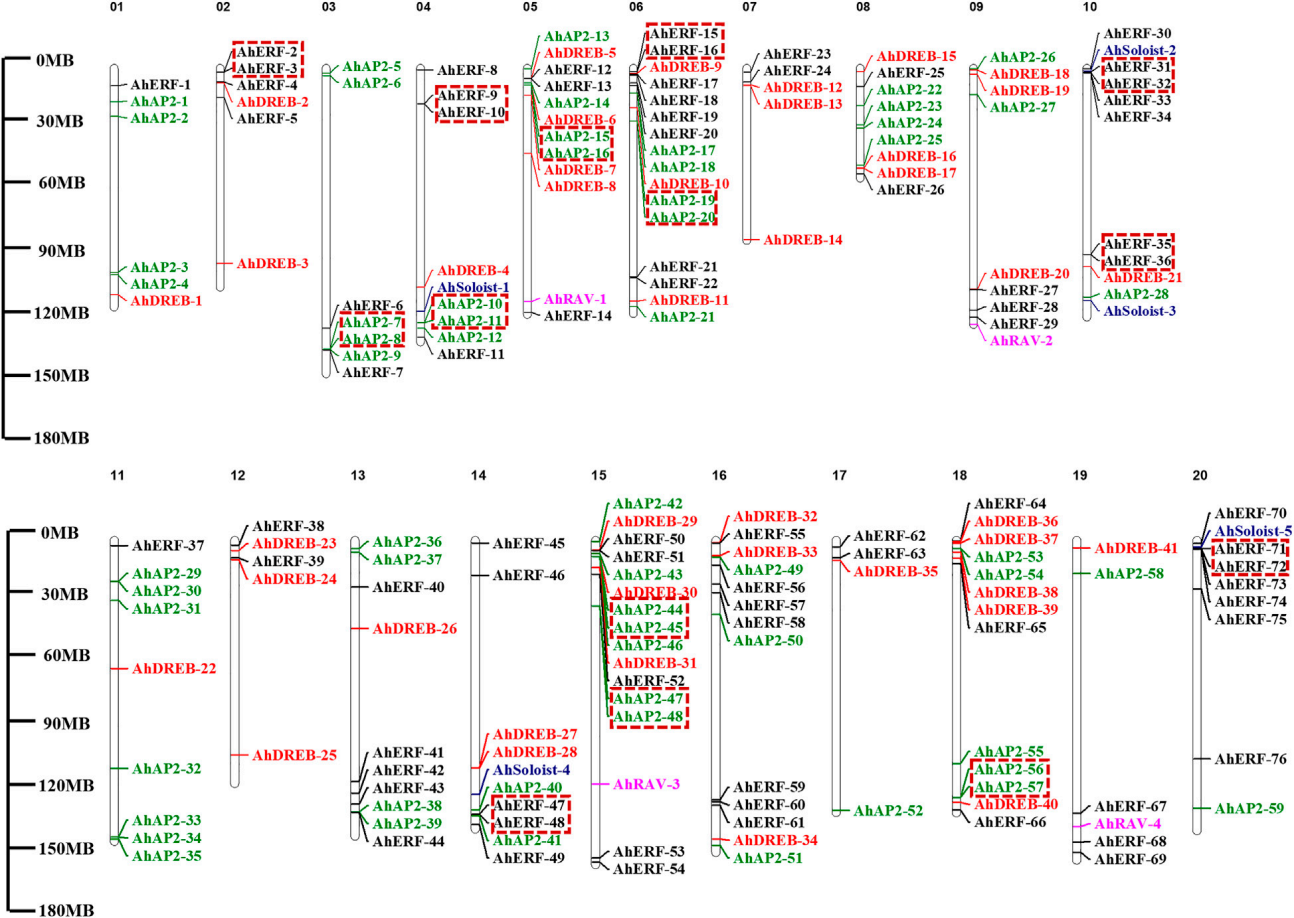
Chromosome mapping and duplication of *AhAP2/ERF* genes. On the right of the chromosome is the gene name. Scale represents a 30-Mb chromosomal distance. Genes in tandem repeats are shown in the red box.

*AP2*/*ERF* genes showed random distribution on the 20 chromosomes of the peanut genome (Figure 3 and Supplementary Table S3). Maximum numbers of *AhAP2/ERF* genes (16 genes) were located on Chromosomes 6 and 15, while Chr17 had the least number of genes (4 genes). Other chromosomes had a random number of allocated *AhAP2/ERF* genes (5–13 genes). Interestingly, most members are distributed at both ends of the chromosome. A similar study on *Arabidopsis* and other species showed consistent findings (Nakano et al., 2006; Guo et al., 2016). This location similarity of genes on chromosomes indicates functional consistency.

### Duplication Events of *AP2/ERF* Genes and Synteny Analysis

**FIGURE 4:**
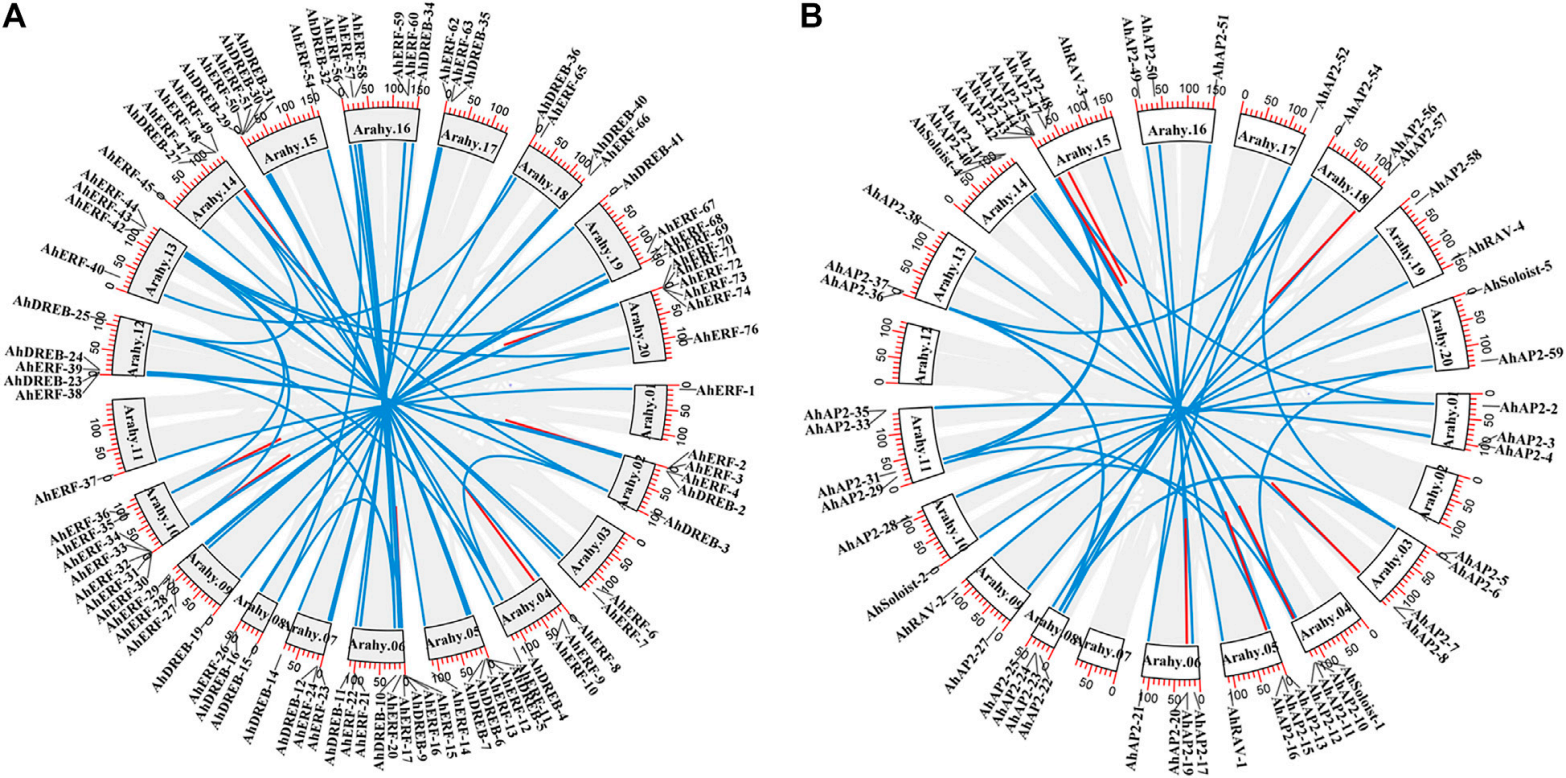
Circos figures for chromosome locations with AP2/ERF duplication links. **(A)** ERF and DREB subfamily duplication links. **(B)** AP2, RAV, and Soloist subfamily duplication links. Blue and red lines indicated segmented and tandem duplicated gene pairs, respectively.

Gene duplication events (segmental or tandem) play a significant role in the expansion and evolution of gene families in plant species (Baloglu, 2014). In total, 99 duplicated gene pairs, which were also named as homoeologous genes, were identified: 14 tandem and 85 segmental duplications (Figure 4). Segmental gene duplication mainly occurred in the *A. hypogaea cv.* Tifrunner genome rather than tandem duplication event (Figure 4 and Supplementary Table S5). No duplication events occurred in group I of the ERF family, whereas more segmental duplication events occurred in other groups of the ERF family, implying that the biggest members of the families might arise from a higher frequency of segmental duplication, when adapting to various environmental shifts. In contrast, tandem duplication has a confined benefaction to the gene family expansion as compared to the segmental duplication. Similar studies on *Arabidopsis*, rice, sorghum (Wang et al., 2011), common bean (Kavas et al., 2015; 2016), and cucumber (Baloglu, 2014) showed compatible findings. In general, segmental duplication might be the main driving force for the *AP2/ERF* gene family expansion in peanut genome.

**FIGURE 5:**
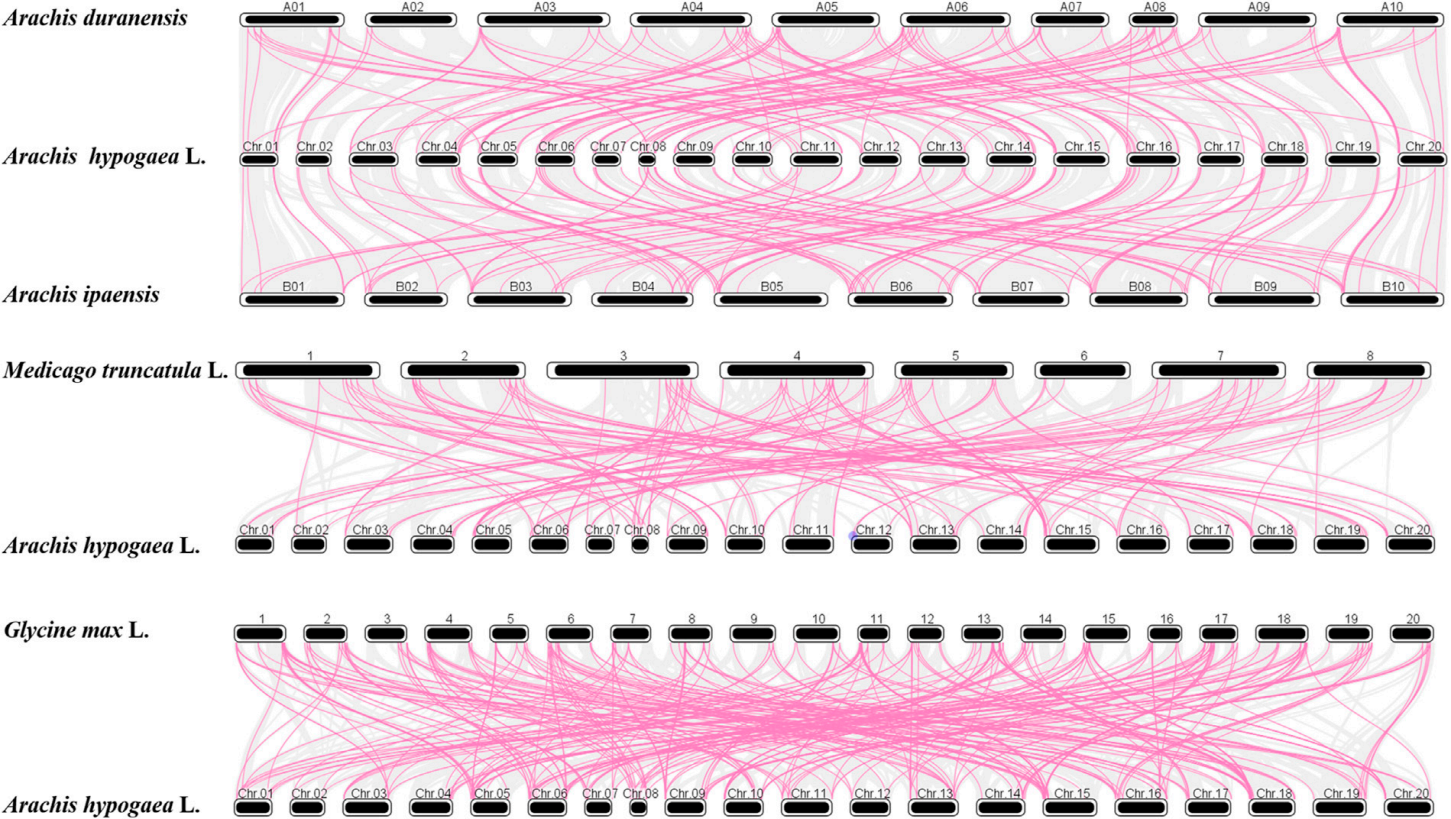
Synteny of AP2/ERF genes in the different genomes of *A. duranensis*, *A. ipaensis*, *Medicago truncatula* L., *Glycine* max L., and *A. hypogaea* cv. Tifrunner.

Moreover, comparative orthologous analysis was conducted among *A. hypogaea cv.* Tifrunner, *A. duranensis*, *A. ipaensis*, *Medicago truncatula* L., and *Glycine max* L. to characterize the evolutionary patterns of *AhAP2/ERF* genes with *Leguminosae* species (Figure 5). In total, 140, 133, 314, and 145 orthologous gene pairs were found with *A. duranensis*, *A. ipaensis*, *Medicago truncatula* L., and *Glycine max* L., respectively (Supplementary Tables S6–S9). The Ka/Ks for segmental duplication was 0.02–0.92 with an average of 0.28, while the ratio of tandem duplication ranged from 0.29 to 0.68 with an average of 0.42 (Supplementary Table S5). These segmental and tandem duplications may occur in ∼3.82–43.68 Mya, respectively. In addition, the Ka/Ks ratio of ortholog gene pairs between *A. hypogaea cv.* Tifrunner and *Medicago truncatula* L. (0.25) and *A. hypogaea cv.* Tifrunner and *Glycine max* L. (0.24) were strongly subjected to pure selection (Lynch and Conery, 2000). The divergence times were 64.07 and 66.44 Mya for *Medicago truncatula* L. and *Glycine max* L., respectively.

### Prediction Analysis of *cis*-Acting Elements With *AhAP2/ERF*

**FIGURE 6:**
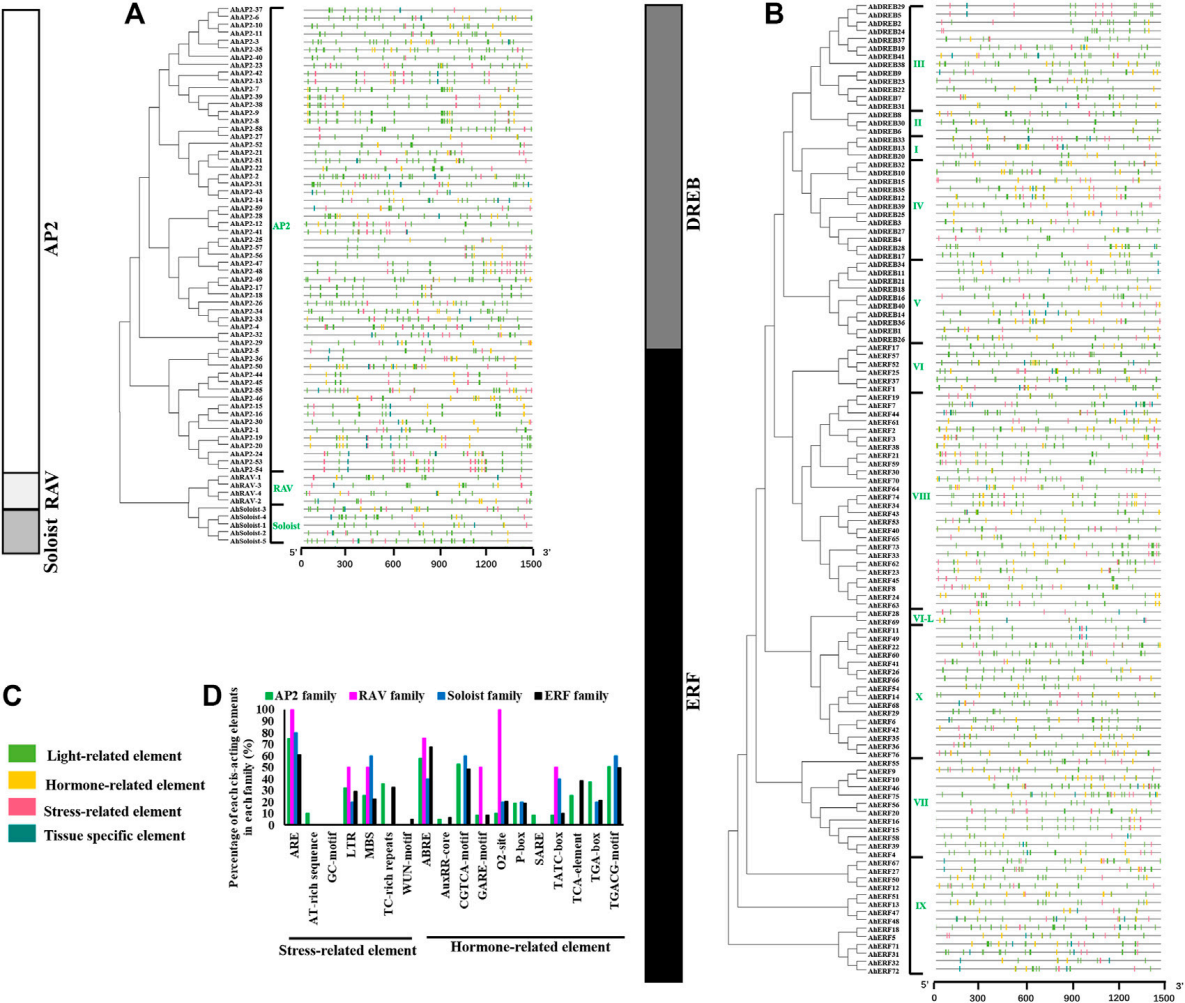
Identification of the *cis*-acting element in the 1.5-kb promoter region of *AhAP2/ERF* genes. **(A)** *cis*-Acting elements of *AhAP2*, *AhRAV*, and *AhSoloist* family genes. **(B)** *cis*-Acting elements of *AhERF* and *AhDREB* subfamily genes. **(C)** The classification and annotation of *cis*-acting elements. Each type of element is represented by a number of colored rectangles. Box length corresponds to element length. Color blocks of different colors represent different family and group members. **(D)** Percentage of each *cis*-acting element in promoter of the *AhAP2/ERF* superfamily.

Specific *cis*-element motifs can be recognized by TFs and participate in gene expression regulation. In order to further study the potential regulatory mechanism of the *AP2/ERF* gene in diversified biological processes, especially in plant drought stress response, the 1.5 kb upstream sequence of the *AP2/ERF* gene translation start site was submitted to the PLANTCARE database to detect cis elements (Cui et al., 2018; Meng et al., 2020). A total of 56 known *cis*-elements (30 light-related elements, 11 hormone-related elements, 8 tissue-specific elements, and 7 stress-related elements) were detected (Figure 6 and Supplementary Table S10). ABRE, AuxRR core, CGTCA motifs, GARE motifs, O2 site, P-box, TATC-box, TCA element, TGA element, and TGACG motifs involved in hormonal responses are found in 63.8, 5.9, 49.2, 9.2%,18.9, 18.4, 10.8, 32.4, 25.9, and 49.2% of *AhAP2/ERF* promoters, respectively. Meanwhile, there are a large number of stress-related elements, including MBS (drought inducibility), TC-rich repeats (defense and stress responsiveness), WUN motif (wound responsiveness), LTR (low-temperature responsiveness), ARE (essential for the anaerobic induction), and GC motif (anoxic specific inducibility) (Supplementary Table S10). Moreover, there was a divergence in the percentage of *cis*-acting elements in promoter regions of various families (Figure 6D). For example, all the RAV family members contained ARE and O2-site elements, whereas 74.6, 80.0, and 60.7% family members of AP2, Soloist, and ERF families possessed ARE elements, and 10.2, 20, and 20.5% of those families exhibited O2-site elements in the promoter region, respectively. Notably, MBS, an important *cis*-element related to the plant drought-inducibility process, was detected in the promoters of 25.4% of AP2, 50% of RAV, 60% of Soloist, and 22.2% of ERF family members. As a major hormone in plant response to drought stress, ABRE possessed 57.6% of AP2, 75% of RAV, 40% of Soloist, and 67.5% of ERF family members. TC-rich repeats, a *cis*-acting element involved in defense and stress responsiveness, were discovered only in the promoters of AP2 and ERF family members. WUN motif, a wound-responsive element, was only detected in the ERF family. The variants in the characterization of *cis*-acting elements implied the functional discrepancy in different families.

What is more, certain *ERF* members in groups III (*AhDREB5/29/41*), IV (*AhDREB15/27/39*), V (*AhDREB21/26*), VI (*AhERF25/52*), VII (*AhERF56*), VIII (*AhERF3/19/30/34/43/53/62/64/65/70/74*), and IX (*AhERF5/31/71/72*) possess a relatively large number of MBS elements in the promoter regions, implying the members’ main role in the regulation of plant drought responsiveness and the function variations among groups (d). In other words, *cis*-acting elements in the same group showed great similarity, indicating the functional conservation in the same groups or clades (Figure 6D).

### Interaction Network Analysis of *AhAP2/ERF* Proteins

**FIGURE 7:**
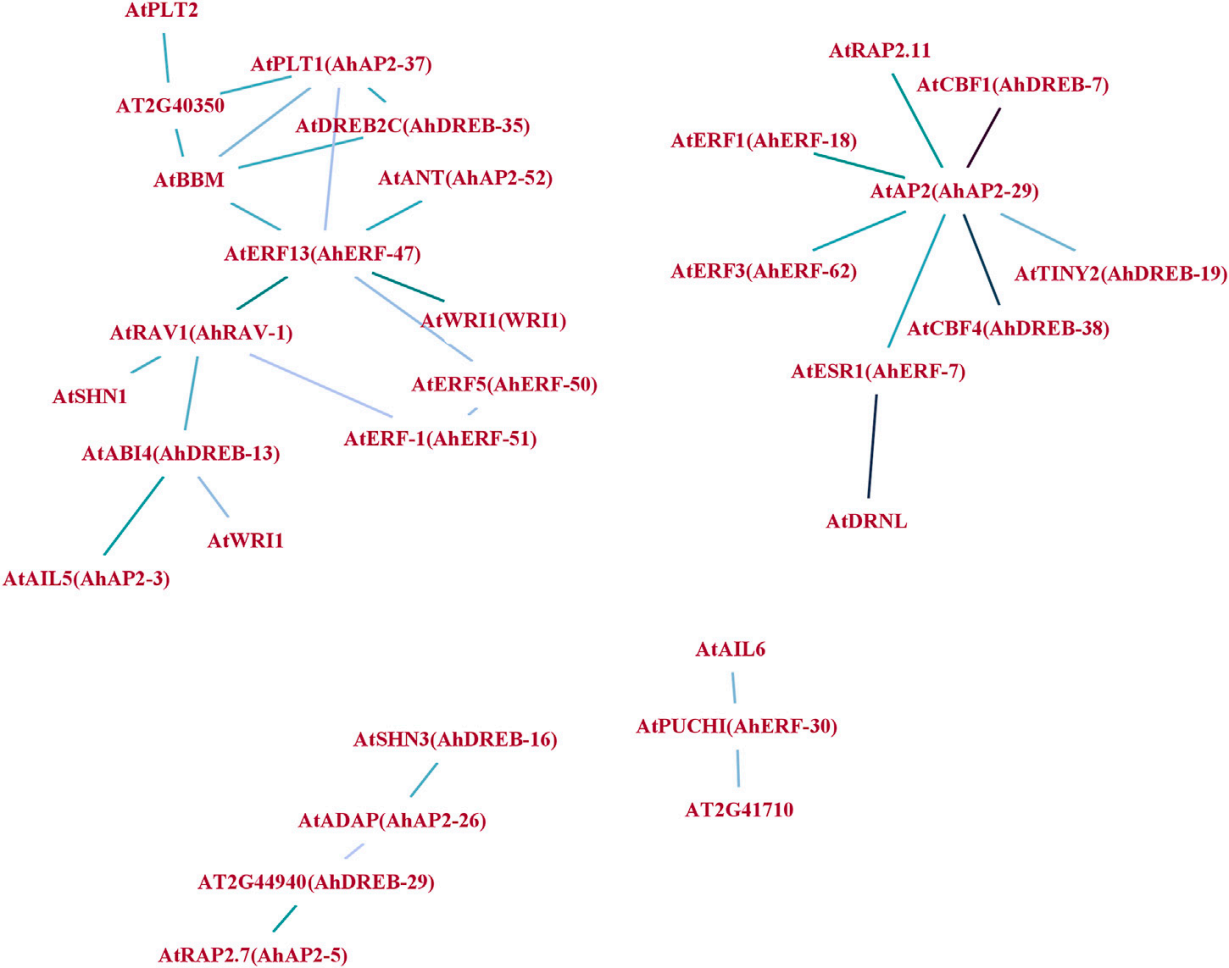
Protein–protein interaction (PPI) network analysis of *AhAP2/ERF* TF proteins. Specific protein interactions between AP2/ERF transcription factors in peanut were determined using String (Szklarczyk et al., 2019). Strong associations are represented by thicker lines.

To understand the synergy among peanut AP2/ERF TFs during their regulatory process, an interaction network was drawn using *Arabidopsis* ortholog genes (Figure 7). A sum of 31 gene pairs with a combined score over zero value was deliberated to have the interaction with others (Supplementary Table S11). *AhAP2-29*, *AhAP2-37*, *AhERF-47*, and *AhRAV-1* had more than three nodes and protein pairs and were involved in more powerful crossing networks, suggesting their core role in peanut. However, other members were only regulated by a few numbers of genes, indicating its less important role in transcriptional level regulation (Figure 7). Interestingly, proteins from different families showed complex interaction. For example, core members *AP2-39* or *RAV-3* interact with genes from *DREB* and *ERF* subfamilies, implying the exitance of functional interaction among subfamilies. These interrelationships will provide a reference for studying the regulatory functions of *AhAP2/ERF* genes in peanut.

### Expression Profiles of *AhAP2/ERF* Genes Under Drought Stress Using qRT-PCR

**FIGURE 8:**
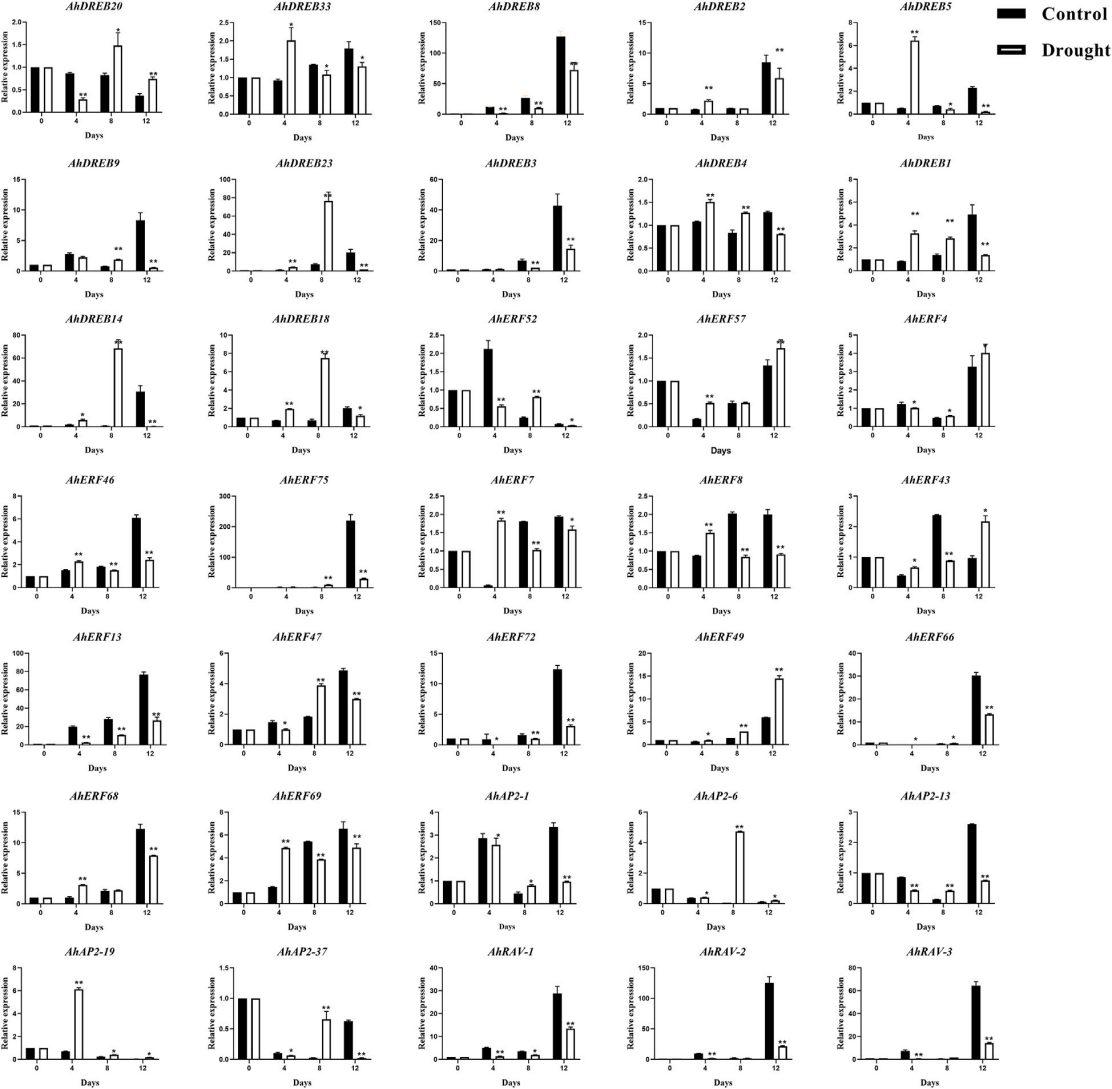
qRT-PCR expression analysis of 35 selected *AhAP2/ERF* genes under drought stress conditions. The expression levels of the untreated (0 h) group were normalized to 1 as a control. Error bars were obtained from three biological replicates. Values are means ± standard errors (SEs) of three independent biological replicates (n = 3). Asterisks indicate a significant difference between the control group and treatment group at each time point as determined by Student’s t-test (*p < 0.05; **p < 0.01).

For better knowing the possible regulatory roles of *AP2/ERF* family genes in peanut response to drought stress, 35 representative genes from AP2 (5), RAV (3), and ERF (27, members from each group) were selected to verify whether their expression levels would be induced under drought stress conditions by qRT-PCR (Figure 8), especially for the ones that possess MBS elements (drought-inducibility), ABRE, TC-rich elements, ARE, and WUN-motif elements in the promoter regions.

The treatment group with no watering was used to stimulate the expression of plant defense genes. The expression analysis of *AhAP2/ERF* genes responsive to drought stress could present useful information to identify their implied role as candidate genes to mitigate drought stress severity. As shown in Figure 8, under the no watering condition, except *AhDREB-3*, *AhDREB8*, *AhERF-4*, *AhERF-13*, *AhERF-66*, *AhERF-72*, *AhERF-75*, *AhAP2-1*, *AhAP2-6*, *AhAP2-13*, *AhRAV-1*, *AhRAV-2*, and *AhRAV-3* exhibited downregulation, whereas the remaining genes were significantly upregulated and subsequently downregulated, at all time points compared with those at 0 h. Furthermore, the peak expression of *AhDREB-1*, *AhDREB-4*, *AhDREB-5*, *AhDREB-33*, *AhERF-7*, *AhERF-8*, *AhERF-69*, and *AhAP2-19* was discovered on the 4th day, but that of *AhDREB-14*, *AhDREB-18*, *AhDREB-20*, *AhDREB-23*, *AhERF-47*, *AhAP2-6*, and *AhAP2-37* was detected at the 8th day. Interestingly, the members in the same group showed semblable expression trends, which indicates the function consistency. Notably, collinear genes *AhDREB-9* and *AhDREB-23*, *AhAP-26* and *AhAP2-37*, *AhRAV-1*, and *AhRAV-3* showed highly similar expression patterns under drought stress treatment, indicating that their biological functions also have a certain similarity.

## Discussion

In plants, AP2/ERF TFs play diverse roles in multiple growth processes and work against environmental factors through transcriptional regulation (Sakuma et al., 2002; Nakano et al., 2006; Woo et al., 2010; Li A. et al., 2017; Yao et al., 2017). Therefore, studying the biological functions and molecular mechanisms of these proteins will facilitate obtaining a deeper understanding of the pathways adapting to environmental pressures during plant growth.

In this study, 185 *AP2/ERF* genes with at least one AP2 conserved domain were identified in the *A. hypogaea cv.* Tifrunner genome through genome-wide analysis. Similar to other plants, all putative *AP2/ERF* superfamily genes were identified as five subfamilies: AP2, ERF, DREB, RAV, and Soloist (Supplementary Table S2). Each subfamily has 59, 76, 41, 4, and 5 members, respectively. In different plants, the numbers of AP2/ERF proteins vary significantly depending on the genome size (eudicot or monocot) (Supplementary Table S12), which may result in gene evolution and duplication. On the other hand, the number of each subfamily follows the regular pattern: the number of the ERF subfamily is the largest, followed by DREB, AP2, and RAV or Soloist (Supplementary Table S12), suggesting that the composition of the AP2/ERF superfamily TFs is highly conserved in plants and may share a common ancestor before separation. Moreover, the largest number of ERF and DREB subfamilies strongly implies its main role in plant growth and development process. The differences in the values of molecular weight (14.46–82.54 kDa) and pI (4.55–10.58) of *AhAP2*/*ERF* suggest the putative differences in *AhAP2/ERF* (Lata et al., 2014). Subcellular localization predicted that *AhAP2/ERF* TFs are mainly localized to the nucleus and thus validates the posttranscriptional regulatory mechanism of the proteins (Karniely and Pines, 2005). Further analysis showed that the AP2/ERFs exhibit certain subfamily characteristics in intron/exon patterns, motif structures, and phylogenetic relationships (Figures 1, 2). This high evolutionary conservation can be used as an important basis for subfamily classification.

Chromosomal mapping showed an uneven distribution of *AhAP2/ERF* genes on 20 chromosomes (Figure 3). There were hot regions or gene clusters on chr06 and chr15. Generally, tandem, large-scale chromosome segmental duplication and transposition were identified as the main evolutionary mechanisms that cause the expansion of the gene family (Lynch and Conery, 2000; Cannon et al., 2004). In total, 14 gene pairs showed tandem duplication and 85 gene pairs revealed segmental duplication, which sustain the overall 8.6% tandem duplication of AP2/ERF in *A. hypogaea cv.* Tifrunner (Zhuang et al., 2019) (Figure 4). The numbers of duplicated gene pairs vary between crops, such as the number of duplication pairs which is 90 in sunflower, 76 in grape, 51 in *Arabidopsis*, 41 in rice, and 11 in dark jute, all of which were lower than in peanut. Hence, this variation in *AP2/ERF* gene numbers in plants might be due to the different duplication events. The microsyntenic analysis of these *AP2/ERF* gene families across the *Leguminosae* family could provide valuable information about their evolution. Our findings demonstrated that a strong association between *AhAP2/ERF* genes of cultivated peanut and wild species was observed (Figure 5). Among them, there were an equal number of pairs of syntenic relationships in the genome of *A. hypogaea cv.* Tifrunner with *A. duranensis* and *A. ipaensis* (Supplementary Tables S6, S7). Notably, most *AhAP2/ERF* genes of *A. duranensis* and *A. ipaensis* might have more than one ortholog in cultivated peanut. These results suggested that cultivated peanut, an allotetraploid plant, likely contained twice the number of AP2/ERF observed for wild-type peanut. The mean Ka/Ks value of peanut with *Medicago truncatula* L. and *Glycine max* L. suggests a purifying selection of *AhAP2/ERF* genes that have undergone great selective constraint and substitution elimination by natural selection (Supplementary Tables S8, S9).

Abiotic stresses do a great harm to the regular growth in peanut at early stages; thus, seedlings at 5 weeks of age were used for drought tolerance (Passioura, 1983; Pierret et al., 2007; Songsri et al., 2008). As an important organ for plants to absorb water and mineral elements, roots directly experience soil drought, and thus, the expression pattern of *AhAP2/ERF* genes under drought stress in roots is essential to clarifying functional divergence (Passioura, 1983; Songsri et al., 2008). The prediction of peanut AP2/ERF protein function by constructing a protein interaction network (Letovsky and Kasif, 2003) proposed the interaction among AP2, ERF, and RAV families, thus implying its interactive function in response to various stresses. For example, *AhAP2-29* showed a strong interaction with *AtRAP2.11*, *AtCBF1*, *AtTINY2*, and *AtESR1*, which are members of the AP2/ERF family that participate in the stress tolerance in *Arabidopsis* (Banno et al., 2001; Novillo et al., 2004; Wei et al., 2005; Min et al., 2012), implying the potential function of AhAP2-29 in peanut response to drought stress. The same phenomenon is discovered in the AP2/ERF family members, including *AhAP2-37*, *AhERF47*, and *AhRAV-1* which are known to function against stress in *Arabidopsis*, thus indicating that there are strong and complex interactions of *AhAP2*, *AhERF*, and *AhRAV* members in peanut response to drought stress. However, under no-watering condition, the expression of *RAVs* was extremely downregulated, perhaps suggesting the indirect role in peanut against drought stress, which is in-line with the previous studies in other crops (Hu et al., 2004; Sohn et al., 2006; Je et al., 2010; Li X.-J. et al., 2015; Wei et al., 2018).

The promoter sequence possesses vital information about gene functional components (i.e., *cis*-acting elements) and reflects potential function of the gene (Kabir et al., 2021). In this study, four distinct types of *cis*-acting elements were found, of which hormone-related and stress-related elements are ones having close relationships with plant stress conditions. Moreover, ABA, as a stress signal, is essential during plant growth and development. It integrates various stress signals and controls downstream stress responses to make plants adapt to various stress environments through uninterrupted adjustment (Tuteja, 2007). Promoter analysis showed that almost all AhAP2/ERF members have ABA response elements, especially ones from AP2, ERF, and DREB subfamilies. Other considerable elements which are related to their function in peanut were TC-rich elements, MBS, and other hormone-related and stress-related elements, which interact in a way. For *ERF*, *DREB*, and *AP2* subfamily genes, it appears that, except *AhDREB-3*, *AhERF-13*, *AhERF-66*, and *AhERF-72*, one owns any *cis*-acting elements of ABRE, TC-rich, MBS, or ARE which may be upregulated by the no-watering treatment, implying the main role of *cis*-acting elements of the promoter in peanut response to drought stress. However, *AhRAV* genes were all downregulated during drought regardless if they are in the promoter region. For the complex interaction of *ERF*, *DREB* , and *AP2* (Figure 7), *AhRAV* may be strongly regulated by other subfamilies. These results suggest that *AhAP2*, *AhERF*, and *AhDREB* genes may play pivotal roles in response to drought stress.

## Conclusion

All in all, a total of 185 *AP2/ERF* genes were identified in the *A. hypogaea cv.* Tifrunner genome and divided into AP2 (59), ERF (76), DREB (41), RAV (4), and Soloist 5) subfamilies. Members in the same family or group shared great similarity of exon–intron structure and conserved motifs. Segmental duplication contributed to the expansion of *AhAP2/ERF* genes, and these duplication pair genes had evolved under strong purifying selection. *cis*-Element analysis suggested that the expression of *AhAP2/ERF* can be regulated by hormones and various environmental factors. Protein interaction predicted complex interaction relationships among or within groups of ERF, DREB, and AP2 members. The expression profile under drought stress by qRT-PCR showed that some *AhAP2/ERF* were significantly upregulated, indicating their potential roles in response to drought stress.

## Data Availability

The datasets presented in this study can be found in online repositories. The names of the repository/repositories and accession number(s) can be found in the article/Supplementary Material.

## Glossary

ABRE: abscisic acid responsiveness element
Ala: alanine
AP2/ERF: APETALA2/ethylene response element-binding factor
Asp: aspartate
AuxRR: auxin responsiveness
ARE: anaerobic response regulatory elements
ARF: auxin response factors
bHLH: basic helix–loop–helix
bZIP: basic leucine zipper
Cs: Cucumis sativus
DREB: dehydration-responsive-element-binding
Elu: glutamic acid
GRF: growth-regulating factor
GARE: gibberellin-responsive element
KDa: kilodalton
LTR: low-temperature response elements
MST: monosaccharide transporter
MEGA: Molecular Evolutionary Genetics Analysis
MEME: Multiple Expectation Maximization for Motif Elicitation
Mya: million years ago
NAC: NAM, ATAF, and CUC
NCBI: National Center for Biotechnology Information
PEG: polyethylene glycol
pI: isoelectric point
qRT-PCR: quantitative real-time
RAV: related to ABI3/VP
SBP: SQUAMOSA promoter-binding protein
SMART: imple Modular Architecture Research Tool
TFs: transcription factors
Val: valine
WUN: wound.
