# Supplementary figures and images for: Genome-Wide Identification and Expression Analysis of AP2/ERF Transcription Factor Related to Drought Stress in Cultivated Peanut (Arachis hypogaea L.)
Source: Front Genet. 2021 Oct 13;12:750761. doi: 10.3389/fgene.2021.750761 (PMC8548641; doi:10.3389/fgene.2021.750761)

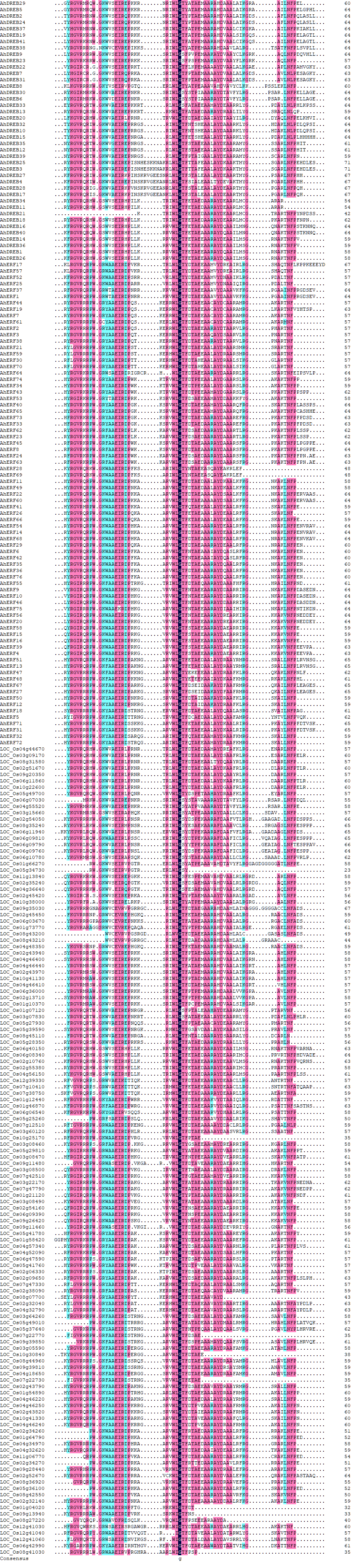

Supplement: Supplementary file 1 [file Image3.TIF]

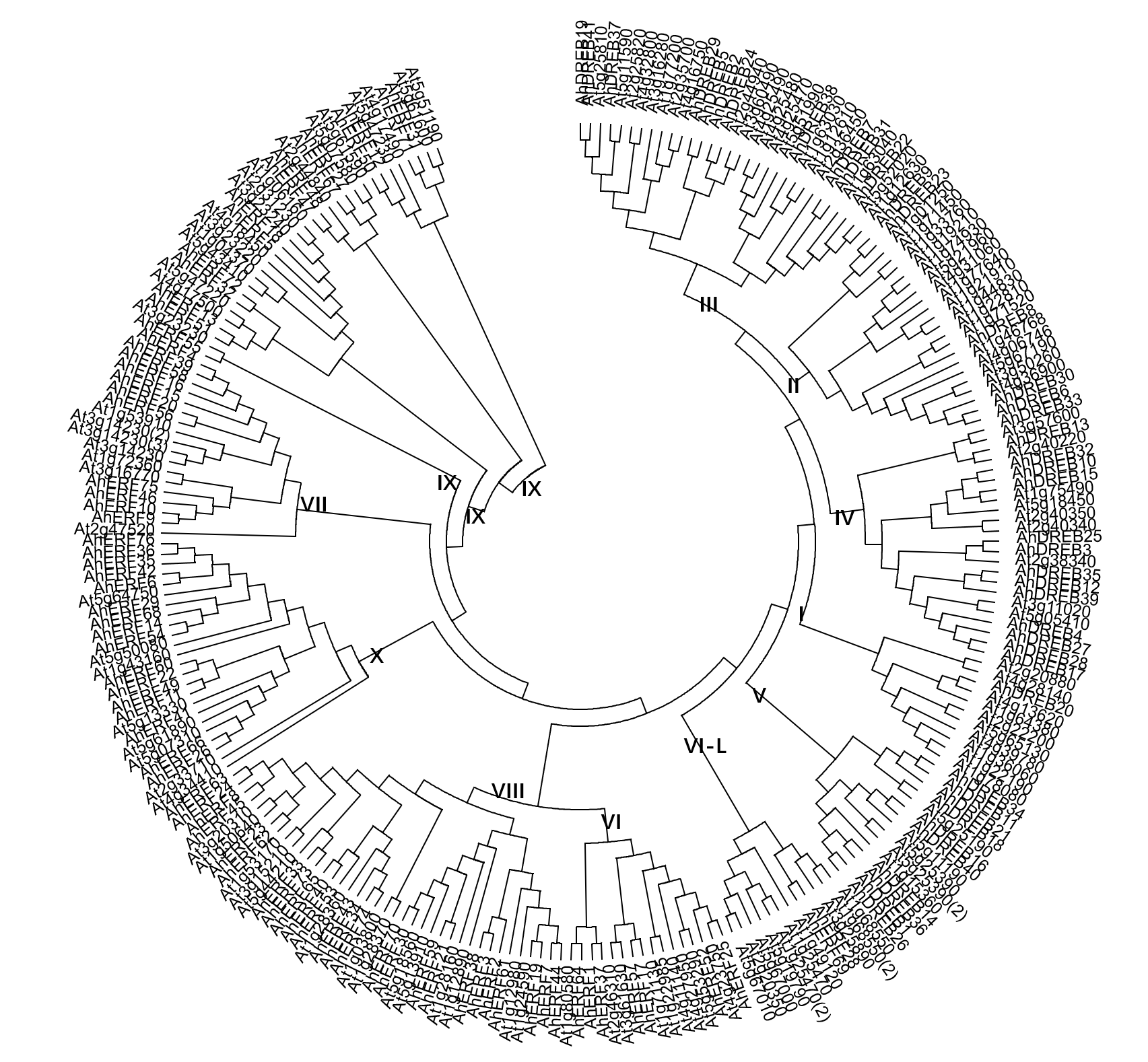

Supplement: Supplementary file 2 [file Image4.TIF]

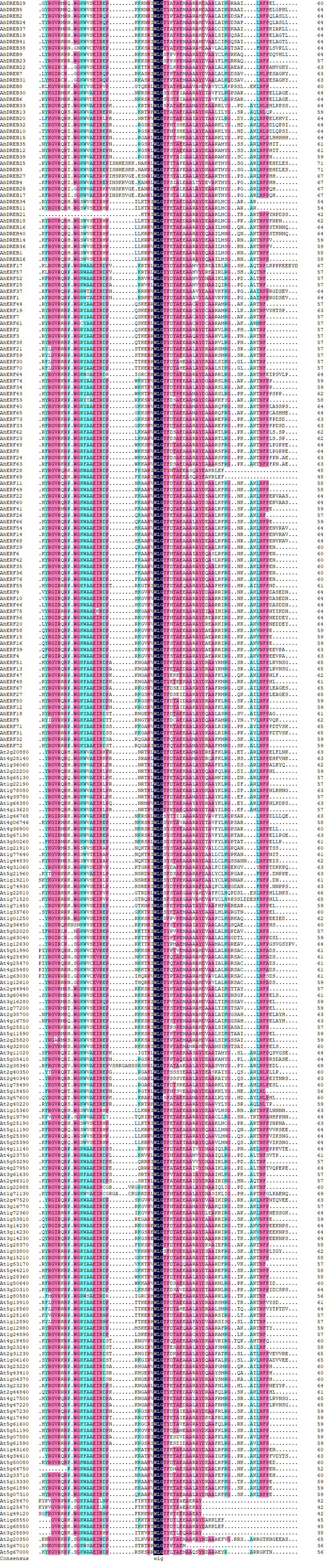

Supplement: Supplementary file 3 [file Image2.TIF]

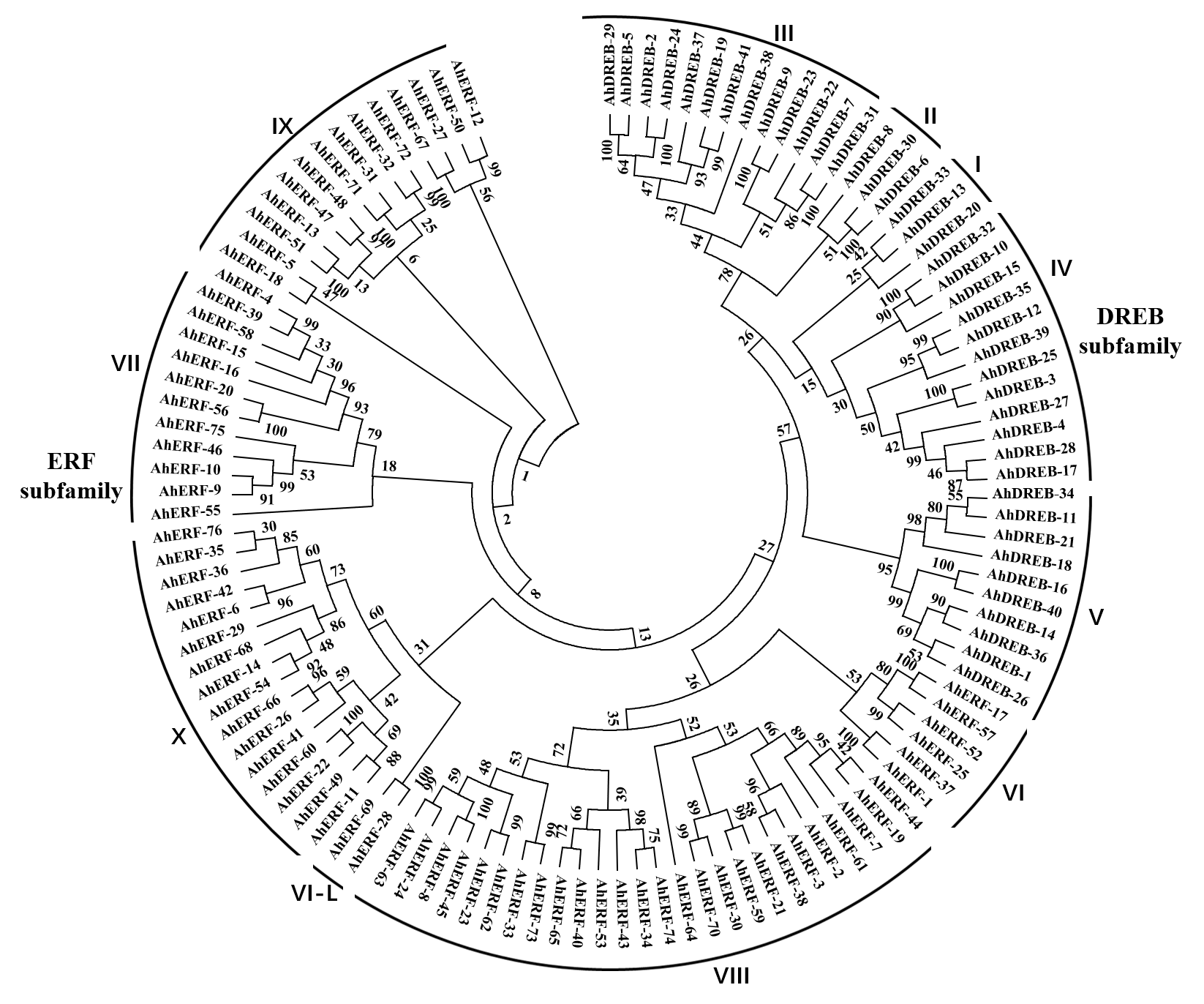

Supplement: Supplementary file 4 [file Image1.TIF]

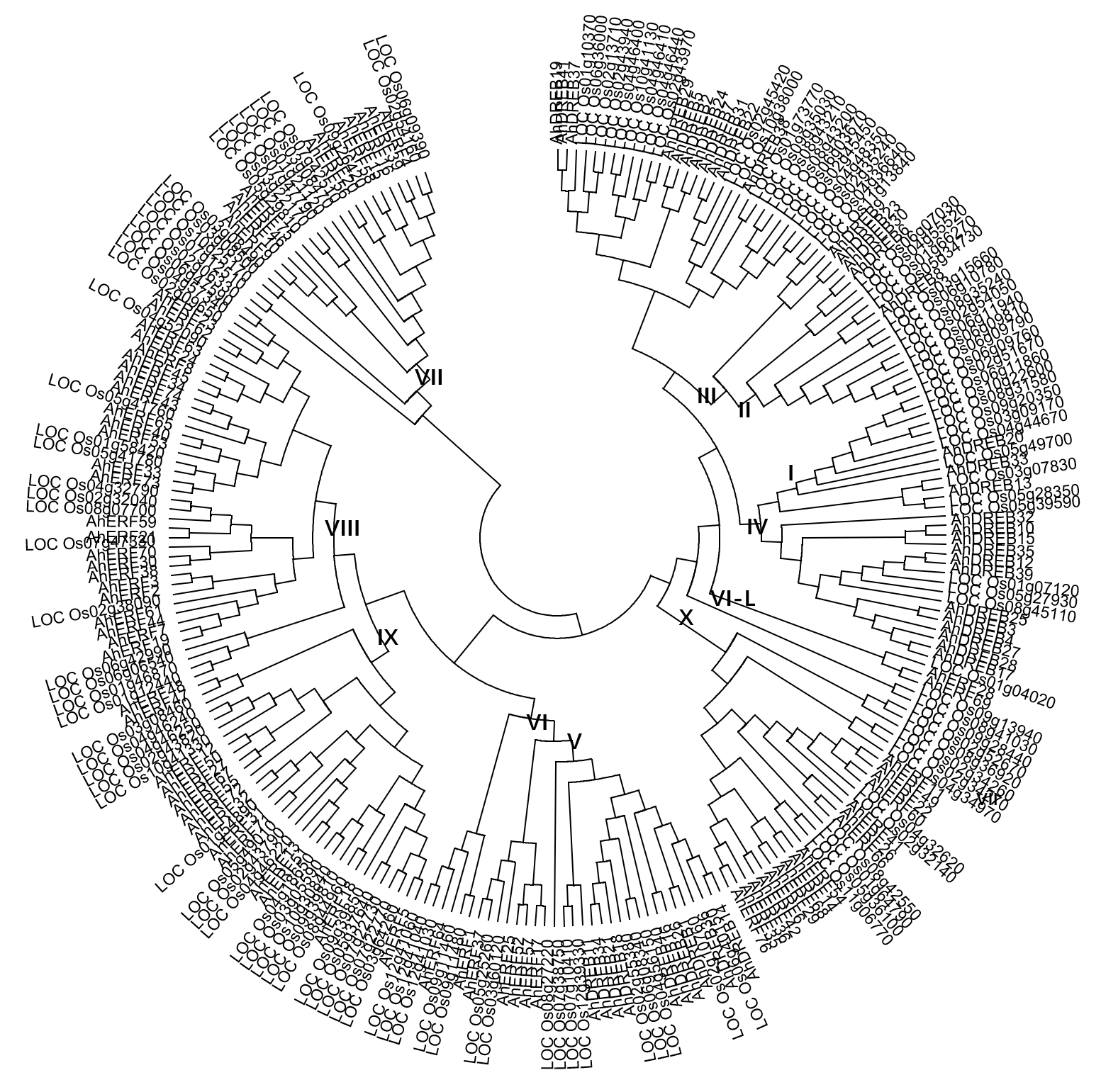

Supplement: Supplementary file 5 [file Image5.TIF]
